# The Yeast Resource Center Public Image Repository: A large database of fluorescence microscopy images

**DOI:** 10.1186/1471-2105-11-263

**Published:** 2010-05-19

**Authors:** Michael Riffle, Trisha N Davis

**Affiliations:** 1 Department of Biochemistry, University of Washington, Seattle, WA 98195, USA; 2 Department of Genome Sciences, University of Washington, Seattle, WA 98195, USA

## Abstract

**Background:**

There is increasing interest in the development of computational methods to analyze fluorescent microscopy images and enable automated large-scale analysis of the subcellular localization of proteins. Determining the subcellular localization is an integral part of identifying a protein's function, and the application of bioinformatics to this problem provides a valuable tool for the annotation of proteomes. Training and validating algorithms used in image analysis research typically rely on large sets of image data, and would benefit from a large, well-annotated and highly-available database of images and associated metadata.

**Description:**

The Yeast Resource Center Public Image Repository (YRC PIR) is a large database of images depicting the subcellular localization and colocalization of proteins. Designed especially for computational biologists who need large numbers of images, the YRC PIR contains 532,182 TIFF images from nearly 85,000 separate experiments and their associated experimental data. All images and associated data are searchable, and the results browsable, through an intuitive web interface. Search results, experiments, individual images or the entire dataset may be downloaded as standards-compliant OME-TIFF data.

**Conclusions:**

The YRC PIR is a powerful resource for researchers to find, view, and download many images and associated metadata depicting the subcellular localization and colocalization of proteins, or classes of proteins, in a standards-compliant format. The YRC PIR is freely available at http://images.yeastrc.org/.

## Background

Understanding a protein's subcellular localization is critical to understanding a protein's role in the cell. The physical location of a protein limits its possible interaction partners and suggests possible biological functions for the protein [1]. The subcellular localization of a protein may be readily assessed by covalently binding it to a fluorescent protein, such as green fluorescent protein (GFP), and viewing the resulting fluorescence by microscopy. The observed pattern and intensity of fluorescence indicate the location and relative quantity of the protein in the cell.

Protein-protein interactions may be assessed via fluorescence microscopy by observing the relative subcellular localization of separate proteins simultaneously tagged with different fluorescent proteins. Proteins with strongly overlapping patterns of subcellular localization are said to colocalize; and this colocalization may indicate similar biological function or possible protein-protein interaction. Interactions may be further characterized by exploiting fluorescence energy transfer (FRET) [2-4], where energy is transferred from the excited fluorophore of a fluorescent protein (bound to the donor protein) to the non-excited fluorophore of a different fluorescent protein (bound to the acceptor protein). Fluorescence of the acceptor protein is then observed where the strength of the signal indicates the efficiency of this energy transfer. The efficiency is partially dependent on the distance between the fluorophores of the two fluorescent proteins and may be used to not only examine whether proteins interact, but to estimate the relative distances between proteins in protein complexes [5].

The data from fluorescence microscopy experiments are typically captured using digital imaging systems attached to fluorescence microscopes and stored as images on disk. Developing computational techniques to automate the analysis of these images is an area of active research [6,7] with direct application to medical imaging as well as basic research. Aberrant subcellular localization has been shown to be associated with certain diseases, including Alzheimer's disease [8] and breast cancer [9]. Algorithms that examine subcellular localizations may be used as a diagnostic aid or as a high throughput tool for finding proteins related to human disease. Additionally, fluorescence microscopy data are used to train algorithms that perform *de novo *prediction of subcellular localization for proteins based on sequence or other criteria.

Researchers developing computational algorithms that analyze fluorescence microscopy images typically require large datasets of images for training and validation of their method. Databases of fluorescence microscopy images have been previously developed, which may aid in this research. The Yeast GFP Fusion Localization Database [10] is a static database containing images for approximately three-quarters of predicted *S. cerevisiae *proteins. YPL.db2 [11] (Yeast Protein Localization database) is a database of fluorescence microscopy images with the aim of annotating the subcellular localization of *S. cerevisiae *proteins. The *Saccharomyces cerevisiae *Morphological Database (SCMD) [12] presents images and phenotypic analysis of 4700 mutant yeast strains. The SCMD includes a large database of fluorescence microscopy images but differs from the YRC Public Image Repository (YRC PIR) in terms of focus. The YRC PIR is a database of images depicting the localization of fluorescently-tagged proteins, whereas the SCMD is a large database of images depicting the phenotype of mutant strains.

The YRC PIR is unique among these database because it is primarily an image database and not a protein annotation database. Where localization databases typically focus on annotating proteins in terms of their subcellular localization by providing chosen examples of images depicting that localization, the YRC PIR aims to provide a large number of images and their associated metadata for many proteins across multiple organisms. Although the YRC PIR may be used to find images depicting the subcellular localization of a particular protein, it is well suited to researchers interested in searching for and downloading large sets of images depicting the localization of particular proteins or categories of proteins.

The YRC PIR expands on existing databases by also offering an intuitive interface to a very large (and growing) database of high-quality and well-annotated images that may be downloaded, along with their associated metadata, as user-defined downloads using standards-compliant formats. In addition to standard subcellular localization data, the YRC PIR contains many images from colocalization and FRET experiments. As the YRC PIR expands, it will grow to include images from many organisms and will accept submissions from researchers wishing to disseminate their data to the public. The YRC PIR should be a valuable tool for researchers developing image analysis and pattern recognition techniques, as well as biologists interested in viewing the subcellular localization for specific proteins or examples of certain subcellular localization patterns. The YRC PIR is freely available at http://images.yeastrc.org/.

## Construction and content

**Figure 1:**
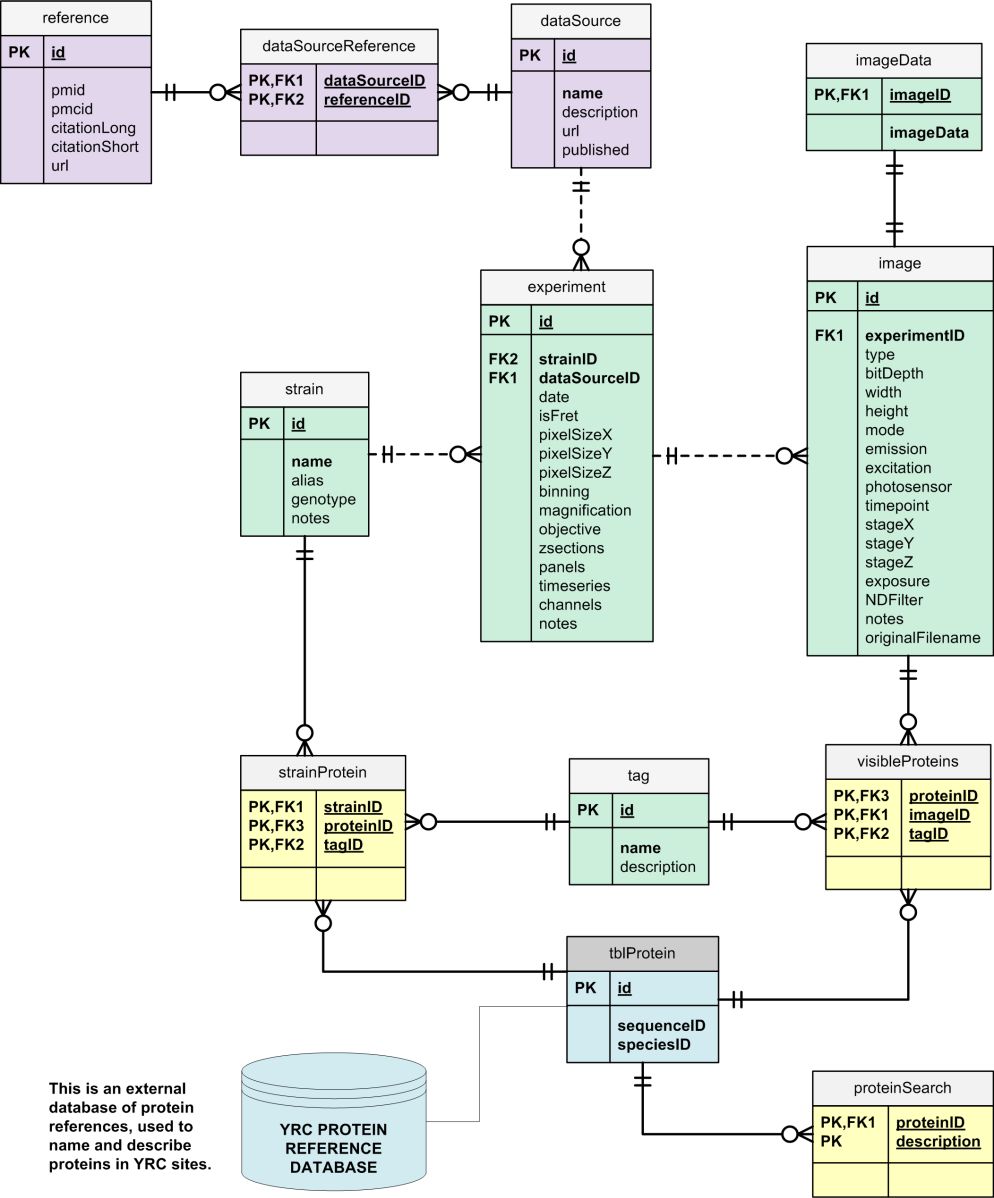
**YRC PIR database schema**. An entity relationship diagram depicting the schema of the YRC PIR database.

The YRC PIR was designed to store and disseminate experimental data generated by disparate microscopy data pipelines. Accordingly, a relational database schema (Figure 1) was developed that captures binary data and metadata common to fluorescence microscopy experiments, which allows for the central storage of data generated by separate laboratories using different software. The current schema design was based on data encountered thus far, and will continue to evolve as new experimental metadata are encountered. The schema was implemented using the MySQL RDBMS http://www.mysql.com/.

The database was initially populated with experimental data from two sources: YRC microscopy data and the Yeast GFP Fusion Localization Database.

### YRC microscopy data

These data existed as proprietary-format binary files generated by the Deltavision microscopy system and resided on multiple servers within the YRC microscopy group. A custom-written Java program was run on a computer with access to the YRC microscopy network that processed these data files and extracted image data, image metadata and experimental metadata and saved the data as XML. The image data existed as raw 12-bit grayscale pixel values, and were converted into uncompressed 16-bit grayscale TIFFs via simple multiplication of pixel values by 16 (2^4^). The XML was transferred to the YRC PIR database server and processed by a custom-written Java program that read the XML input and saved the data to the relational database schema.

These data comprise 516,456 images from 79,428 separate data files. The data depict 68 proteins from *Saccharomyces cerevisiae *and *Schizosaccharomyces pombe*, mostly related to mitosis. The data include protein subcellular, protein colocalization and FRET image data.

### Yeast GFP Fusion Localization Database

These data existed in multiple forms. The experimental metadata were contained in a MySQL database. The DIC (visible light) and DAPI (blue, DNA-stained) channel images existed as 16-bit grayscale PNG files. The GFP and RFP channel images existed as 48-bit color PNG files, with fluorescence depicted in the green and red channels, respectively. In order to preserve data consistency in the YRC PIR database, all PNG files were converted to 16-bit grayscale TIFF images using the ImageMagick http://www.imagemagick.org/ software package with the command line command: " fmogrify -format tiff -compress none -type Grayscale *.png".

The MySQL database and resulting TIFF images were processed and inserted into the YRC PIR relational database schema by a custom-written Java program. These data comprise 14,526 images from 4,842 experiments. The data depict 4,176 proteins, all from *Saccharomyces cerevisiae*. The data include protein subcellular localization and colocalization images.

### Web Server

A web interface for searching and downloading the data was written in Java and JSP using the Apache Struts web application framework running on the Apache Tomcat servlet container and Apache httpd web servers.

### Downloaded images

All images downloaded via any of the methods for downloading "raw" data from the YRC PIR are formatted as OME-TIFF files. The Open Microscopy Environment (OME) defined OME-TIFF such that it is fully compatible with standard TIFFs and, consequently, software that is able to read and write TIFFs. OME-TIFF files include experimental and image metadata by incorporating OME-XML[13] into one of the TIFF headers. The TIFFs are all uncompressed, 16-bit grayscale images.

## Utility and Discussion

**Figure 2:**
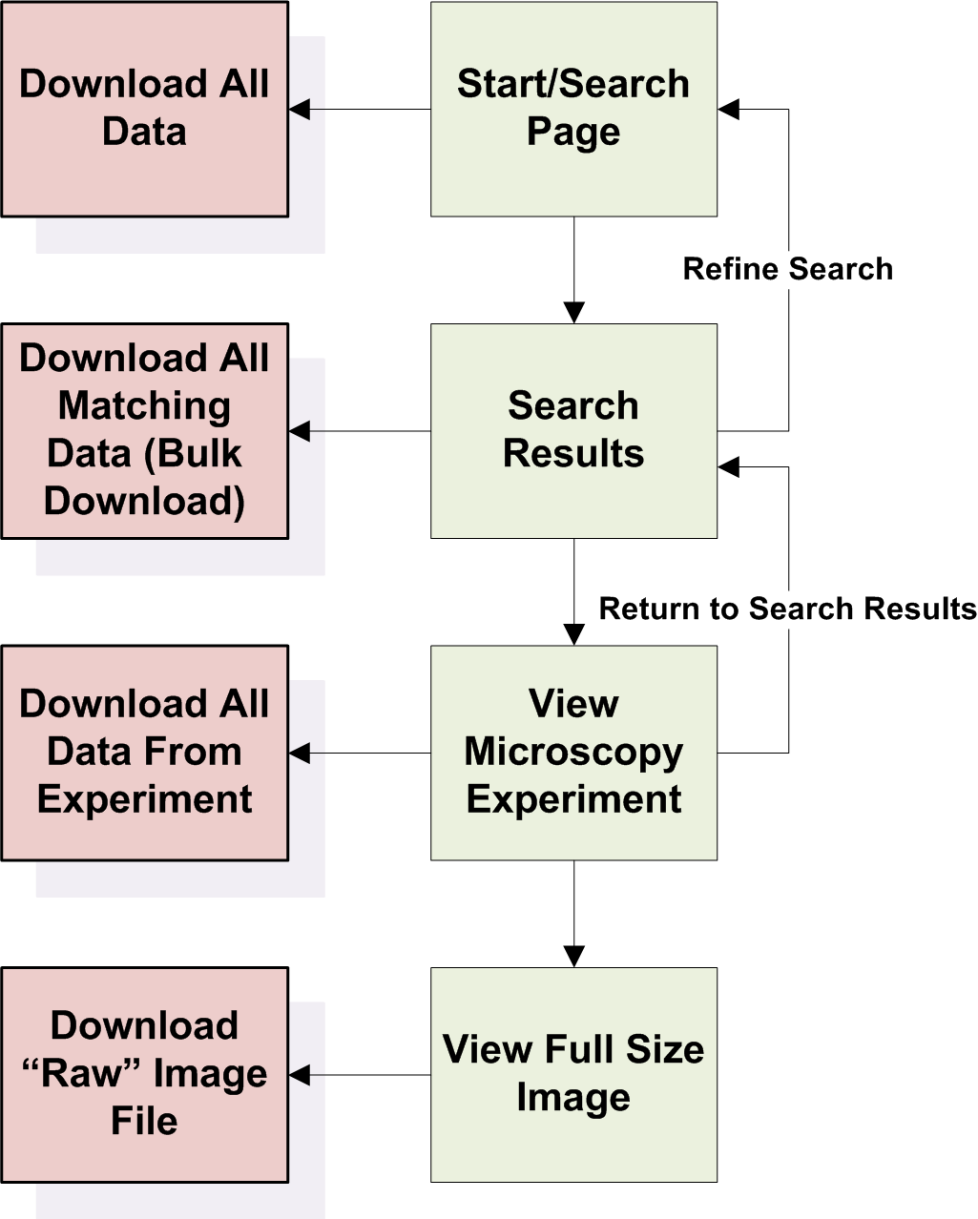
**YRC PIR web site design overview**. The YRC PIR provides four ways for downloading raw data, each linked to four levels of scope for viewing data. First, all the data may be downloaded at once from the data download page. Second, all data matching a given search may be downloaded. Third, all data from a given experiment may be downloaded. And fourth, specific images may be downloaded.

The YRC PIR's design is focused on searching, viewing results, refining searches and downloading or viewing pertinent raw data (Figure 2). An extensive user guide and FAQ were written and are available at the YRC PIR web site. Additionally, all data in the YRC PIR may be downloaded via the download page.

**Figure 3:**
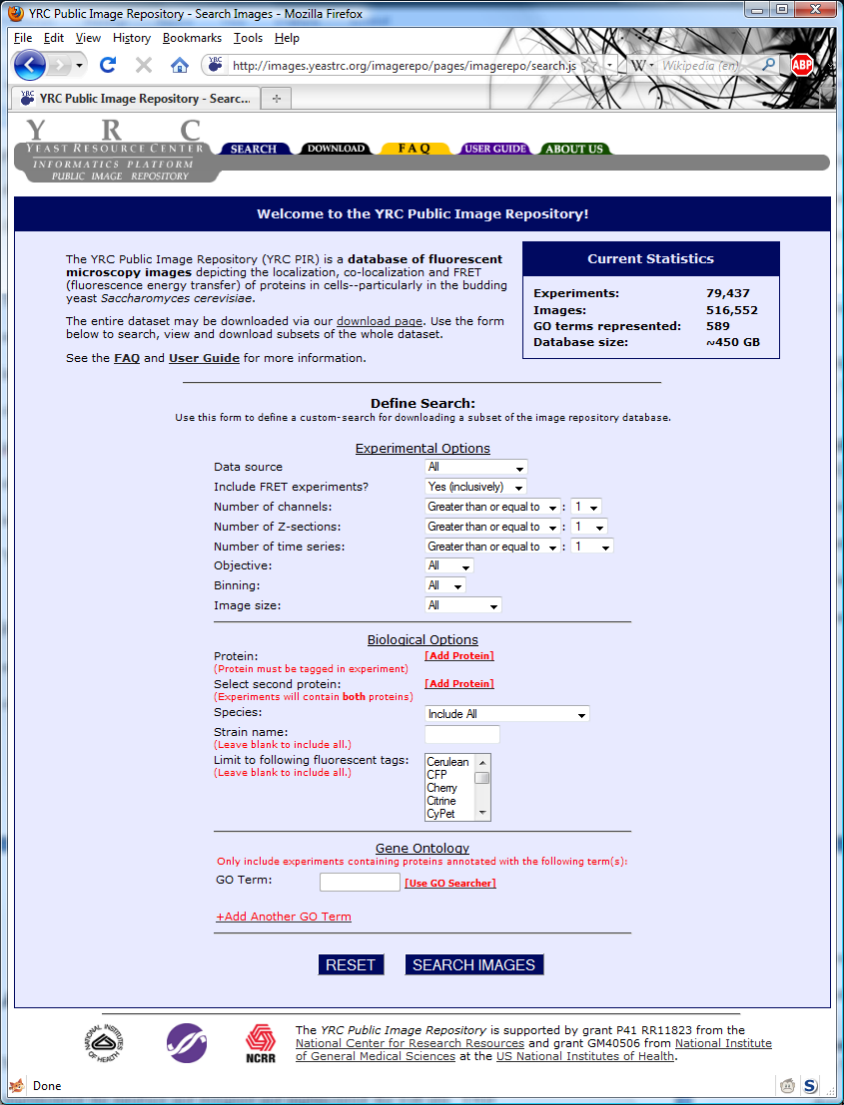
**YRC PIR search page**. The YRC PIR search page contains many parameters for searching and eventually downloading microscopy data.

A web interface was constructed for searching microscopy experiments (Figure 3). From this form, a user may search microscopy experiments based on the source of the data, Gene Ontology (GO) [14] annotations of tagged proteins, names of tagged proteins, strain names, fluorescent tags used, image sizes, inclusion of fluorescence resonance energy transfer (FRET) experimental data, inclusion of differential interference contrast (DIC) images, and other experimental parameters.

**Figure 4:**
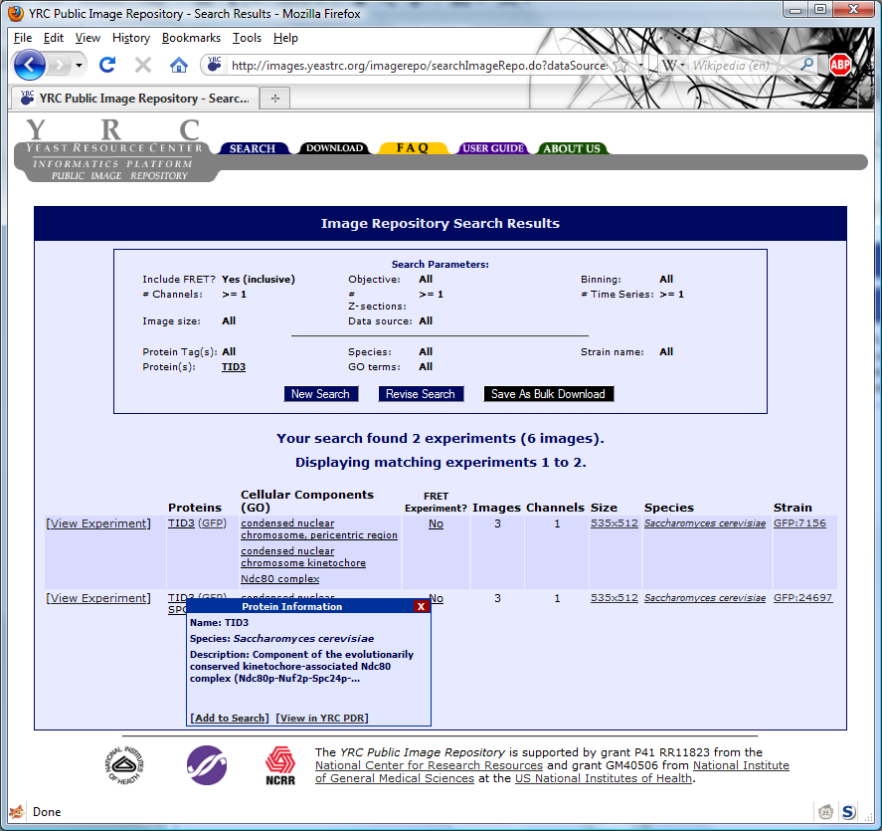
**YRC PIR search results page**. The YRC PIR search results display all matching projects as a table with summary information for each experiment. The search may be constrained by clicking on summary information.

The search results consist of a synopsis of the search parameters and a table of microscopy experiments that match the search parameters (Figure 4). Each row in the table represents a matched experiment, and the columns include data that provide an overview of the experiments, including tagged proteins, GO annotations of tagged proteins, whether or not it is a FRET experiments, the number of images in the experiment, the number of wavelength channels used in the experiment, size of the images, the species used in the experiment and the name of the strain used in the experiment.

The search may be constrained by most of the values present in these search results by clicking on the value in the table. For example, clicking on the name of a strain from one of the listed experiments will re-search the database with the added constraint of that strain name. Clicking the value for fluorescent tag used (e.g., CFP or YFP), image size and species will also constrain the current search. Hovering the mouse cursor over protein names, GO terms, or whether or not FRET was used in the experiment will produce a "pop up" tool tip that provides a description of that value (such as a description for that GO term) and provides links for adding that constraint to your search or learning more about that protein or GO term by visiting the YRC Public Data Repository (YRC PDR) [15]. The YRC PDR is a separate database developed by the authors that, in contrast the YRC PIR, focuses on annotating proteins with disparate types of experimental data and with names, descriptions, and annotations mirrored from many other large, public databases.

From this search results page, the user may refine their search (by clicking "Refine Search"), download all data that matches the current search (by clicking "Save as Bulk Download") or view the images and metadata associated with any experiment (by clicking the "View Experiment" link associated with the desired experiment). The "Save as Bulk Download" option prompts the YRC PIR system to create an archive of all images and metadata that match the current search and then notify the user (at the email address specified) that their data is ready to be downloaded. The organization of this file is described extensively in the User Guide available at the YRC PIR web site.

**Figure 5:**
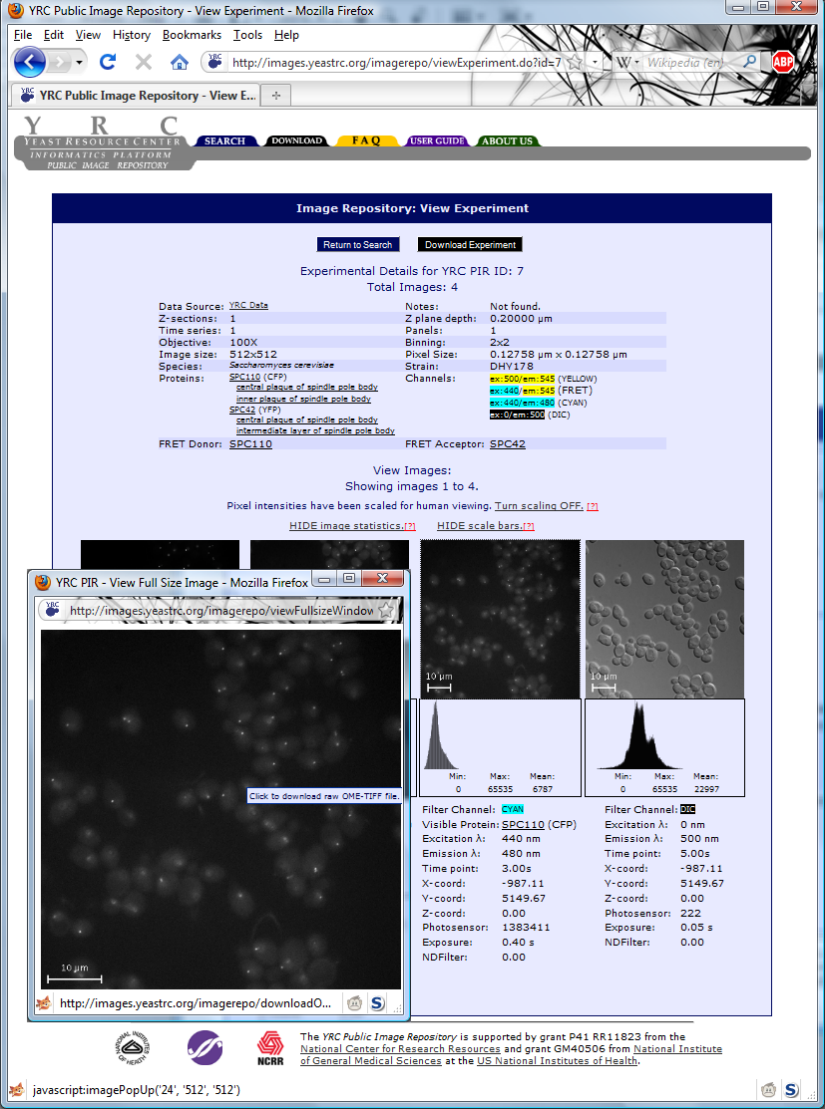
**YRC PIR experiment view page**. The YRC PIR experiment view page contains experimental metadata, thumbnails of microscopy images and their associated metadata. The thumbnails may be clicked on to retrieve full size images. The full size images may be clicked on to download a raw OME-TIFF version of the image.

The "View Experiment" link takes the user to a page listing all experimental metadata and all images (and their associated metadata) associated with the experiment (Figure 5). From here the user may download all metadata and images associated with this specific experiment by clicking the "Download Experiment" button. The experimental metadata include: Data source, experimental notes, the number of Z-sections, Z plane depth, the number of time series, the number of panels, the power of the objective used, the binning applies to the captured images, the pixel size of the images (image size), the physical size represented by each pixel (pixel size), the species, the strain name, the proteins tagged in their experiment (including the protein tag used, and GO terms associated with those proteins), the color channels used in the experiment and the FRET donor and acceptor (if it is a FRET experiment). Further description and definitions of these data can be found in the FAQ at the YRC PIR web site.

Below the metadata, the images associated with the experiment are shown as thumbnails along with accompanying metadata. By default, the images have their intensity values scaled so that the fluorescence is easier to observe in the web site. This effect may be disabled by clicking "Turn scaling OFF," just above the images. Also by default, a scale bar is present in each image that depicts the physical scale of objects in the image. This may also be disabled by clicking "HIDE scale bars." Very simple image statistics may be displayed by clicking "SHOW image statistics". These statistics include minimum, maximum and mean pixel values in each image, along with a histogram depicting the distribution of pixel values.

The displayed thumbnails are in PNG format, and are converted and resized on-the-fly from 16-bit grayscale TIFFs stored in the database. Clicking on a thumbnail will cause a full-size version of the image to appear in a pop up window, also in PNG format. Clicking on the full-size image will trigger a download of a "raw" version of that image. As with all image data that is available for download from the YRC PIR, the "raw" version will be an unmodified (original pixel values, size, and no scale bar) and uncompressed OME-TIFF file.

Metadata are displayed for each image in the experiment. These include the name of the tagged protein and the name of the covalently-bound fluorescent protein that are visible in the image. The pattern of fluorescence in these images indicates the location of the tagged protein in the cell, and the strength of the signal is proportional to the amount of tagged protein present. For images of FRET channels, the name of the donor and acceptor proteins and their associated fluorescent proteins are displayed instead. The fluorescent protein bound to the donor protein has been excited and fluorescence from the acceptor protein is visible in the image. In these images, the intensity of this signal is a measure of the potential distance between the two fluorescent proteins and, by extension, between the donor and acceptor proteins. For images taken using differential interference contrast (DIC), whole cells are shown using transmitted light and no data describing the localization of a protein are shown.

Additional metadata include the excitation and emission wavelengths of light used to excite and detect fluorescence from the fluorescent proteins in the image. (Except in the case of DIC images where the excitation and emission wavelengths are not meaningful.) The filter channel categorizes images as DIC, FRET or standard color channels. The photosensor is the measured brightness of the lamp. Exposure describes how long the shutter was open. NDFilter describes the level of neutral density filter applied to the image. More descriptions of the metadata are available in the FAQ available at the YRC PIR web site.

Further work on the YRC PIR will include analysis tools to be available on the experimental view page, such as creating RGB merged-color images of the different channels in the experiment, the ability to perform FRET analysis and co-localization analysis, and other analysis tools common to fluorescence microscopy studies. Additionally, a web interface will be developed for users in the general community to upload data from their own experiments into the YRC PIR. The YRC PIR will be a platform for data sharing, suitable for collaboration and distributing data associated with microscopy publications.

## Conclusions

To our knowledge, the YRC PIR is the largest public database of fluorescence microscopy images currently available. To make this large dataset manageable to users, an emphasis was placed on search options and intuitive data view pages that lead to multiple options for downloading raw data. This makes the YRC PIR a valuable tool for computational biologists interested in defining and downloading datasets specific to their research and to cellular and molecular biologists interested in viewing the subcellular localization of particular proteins or viewing examples of subcellular localization patterns.

## Availability and requirements

The YRC PIR is freely available to all at http://images.yeastrc.org/.

## List of abbreviations

YRC PIR: Yeast Resource Center Public Image Repository; GFP: Green Fluorescent Protein; FRET: Fluorescence Resonance Energy Transfer; DIC: Differential Interference Contrast; RFP: Red Fluorescent Protein; OME: Open Microscopy Environment; GO: Gene Ontology; CFP: Cyan Fluorescent Protein; YFP: Yellow Fluorescent Protein.

## Authors' contributions

MR drafted the manuscript and developed the data parsing software, designed and implemented the database, and designed and implemented the web site. TND provided input during all phases of development and participated in drafting and revising the manuscript. All authors have read and approved the manuscript.
